# Knockdown of Amyloid Precursor Protein: Biological Consequences and Clinical Opportunities

**DOI:** 10.3389/fnins.2022.835645

**Published:** 2022-03-14

**Authors:** Rebecca M. C. Gabriele, Emily Abel, Nick C. Fox, Selina Wray, Charles Arber

**Affiliations:** ^1^Department of Neurodegenerative Disease, Queen Square Institute of Neurology, University College London, London, United Kingdom; ^2^UK Dementia Research Institute at University College London (UCL), Queen Square Institute of Neurology, London, United Kingdom

**Keywords:** amyloid precursor protein (APP), amyloid-beta, Alzheimer’s disease, CRISPR, antisense oligonucelotides

## Abstract

Amyloid precursor protein (*APP*) and its cleavage fragment Amyloid-β (Aβ) have fundamental roles in Alzheimer’s disease (AD). Genetic alterations that either increase the overall dosage of *APP* or alter its processing to favour the generation of longer, more aggregation prone Aβ species, are directly causative of the disease. People living with one copy of *APP* are asymptomatic and reducing *APP* has been shown to lower the relative production of aggregation-prone Aβ species *in vitro*. For these reasons, reducing APP expression is an attractive approach for AD treatment and prevention. In this review, we will describe the structure and the known functions of APP and go on to discuss the biological consequences of APP knockdown and knockout in model systems. We highlight progress in therapeutic strategies to reverse AD pathology via reducing *APP* expression. We conclude that new technologies that reduce the dosage of *APP* expression may allow disease modification and slow clinical progression, delaying or even preventing onset.

## Introduction

Alzheimer’s disease (AD) is characterised pathologically by the accumulation and extracellular deposition of Amyloid-β (Aβ) peptide into amyloid plaques, as well as intraneuronal aggregates of tau protein and progressive neurodegeneration (Hardy and Allsop, 1991).

Our understanding of the central role of Aβ in the pathogenesis of AD has been informed by studies of autosomal dominantly inherited (or familial) AD. Familial AD (fAD) is caused by mutations in three genes: amyloid precursor protein (*APP*), presenilin 1 and presenilin 2 (*PSEN1*/*PSEN2*) (Goate et al., 1991; Levy-Lahad et al., 1995; Sherrington et al., 1995). *PSEN1* and *PSEN2* encode the catalytic subunit of the γ-secretase complex that is involved in processing of the APP protein to produce Aβ. Pathogenic mutations in *PSEN1* and *PSEN2* destabilise γ-secretase processing of APP, causing the release of longer and more aggregation prone species of Aβ (Szaruga et al., 2017). The relative levels of longer (such as Aβ42) to shorter species (such as Aβ40) is not only a marker of pathogenicity in fAD but also determines age at clinical onset (O’Connor et al., 2021). fAD mutations cause disease with almost complete penetrance (Ryan et al., 2016), typically resulting in an age of onset decades before the sporadic form of AD.

Crucially relevant to this review, genetic alterations that increase the dosage of *APP* also lead to Alzheimer’s pathology and to early onset clinically manifest AD. Trisomy of chromosome 21 in Down’s syndrome (DS) leads to three copies of *APP* and a concurrent high incidence of Alzheimer’s disease in DS patients (Wisniewski et al., 1985). Additionally, duplications (Rovelet-Lecrux et al., 2006; Sleegers et al., 2006) and triplications (Grangeon et al., 2021) of the local *APP* gene territory cause fAD. In contrast, people living with one functional copy of *APP* are asymptomatic (Klein et al., 2016) and mutations that reduce amyloidogenic processing of APP can be protective against AD (Jonsson et al., 2012). Models with repaired APP mutations show normalised Aβ profiles (Kwart et al., 2019), and importantly, reducing the concentration of APP substrate enables more complete Aβ processing by γ-secretase, thereby lowering the relative production of longer Aβ species such as Aβ42 (Ye et al., 2007).

There remain no therapies that can prevent, slow or reverse AD. 2021 saw US food and drug administration (FDA) approval of aducanumab, the first potential disease modifying therapy for AD based on evidence of an ability to clear amyloid pathology (Sevigny et al., 2016). Aducanumab is a monoclonal antibody that targets aggregated forms of Aβ, with particular ability to neutralise Aβ seeds (Uhlmann et al., 2020). However, this approval has been controversial largely because clinical efficacy has not been demonstrated. Irrespective of this approval, finding therapies that show clinically meaningful benefit and can slow or prevent AD remains a global priority—and multiple approaches may be necessary.

There are different hypotheses around pathogenesis of AD (Hunter and Brayne, 2018) and the relative merits of amyloid and tau as therapeutic targets are debated. However, the fact that increased APP dosage is causative of familial forms of the disease supports the idea that reducing APP expression could reduce the risk of dementia and potentially slow progression–at least for fAD. This hypothesis works on the assumption that APP (acting through Aβ) is critical to the initiation of the disease and is likely to be upstream of other disease-associated processes such as tau, microglia/inflammation and metabolic alterations (Hunter and Brayne, 2018). In this review we will describe the biological roles of APP and discuss the consequence of genetic knockdown and knockout in model systems. We will then provide an overview of current therapeutic approaches targeting APP knockdown and discuss the relative biological merits of these approaches.

## Amyloid Precursor Protein

### Structure and Expression

APP was first cloned in 1987 (Kang et al., 1987), prior to the discovery of genetic polymorphisms associated with familial AD (Hardy and Allsop, 1991). Since that time, our understanding of the structure of *APP* gene and protein has progressed considerably.

**FIGURE 1:**
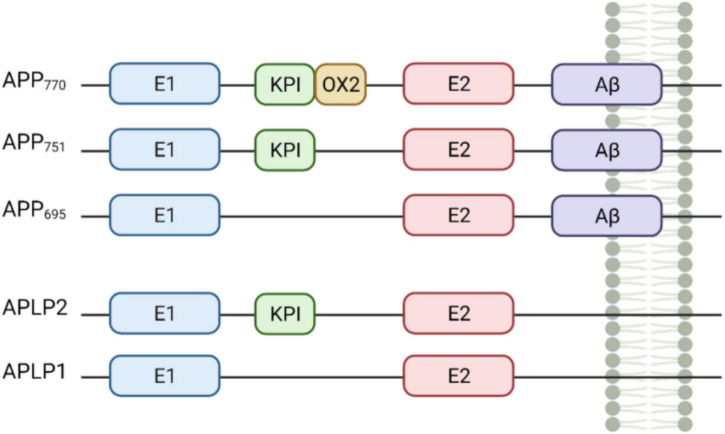
Domains of amyloid precursor protein (APP) and the APP-like proteins (APLP) protein family members. APP and its mammalian homologues APLP1 and APLP2 share similar domain architecture including the E1 and E2-domains, which potentially drive dimerisation. APP770, APP751, and APLP2 are characterised by the Kunitz type protease inhibitor domain (KPI) upstream of E2. APP770 includes also the OX2 domain. Both APP and APLPs contain a transmembrane domain (TMD) but only APPs have the Aβ sequence (purple). Created with BioRender.com.

APP and the APP-like proteins (APLP) APLP1 and APLP2 are all encoded by genes in the same gene family (Shariati and De Strooper, 2013). The *APP* gene resides on chromosome 21q and contains 18 exons. Structurally, APP is a 110–130 kDa type 1 transmembrane glycoprotein, consisting of a single-pass transmembrane domain, a large extracellular *N*-terminal domain and a shorter cytoplasmic *C*-terminal tail (Chen et al., 2017). APP and the APLPs have similar structures, sharing conserved regions including the *C*-terminal intracellular domain and the E1 and E2 domains within the extracellular domain (Figure 1). Importantly, unlike APP, the APLPs lack the Aβ sequence, meaning they cannot give rise to the Aβ peptide associated with Alzheimer’s disease (Müller et al., 2017).

Differential splicing of exons 7 and 8 of APP results in the generation of three major APP isoforms—APP_695_, APP_751_, and APP_770_ (Nalivaeva and Turner, 2013). In the brain, APP_695_ is the major isoform and is predominantly found in neurons. APP_751_ and APP_770_ are expressed at lower levels and are found mostly in astrocytes (Smith et al., 2011) as well as in fibroblasts and peripheral tissues (Haass et al., 1991; LeBlanc et al., 1991). Microglia and other blood cells express an additional isoform that lacks exon 15 of APP (Banati et al., 1993). It is unclear how different isoforms alter the physiological function of APP in different contexts, however, neuronal APP_695_ lacks the KPI and Ox-2 domains, which are involved in protein-protein interaction, suggesting cell-specific APP biology (Menéndez-González et al., 2006).

Expression of APP mRNA in mice has been noted early in development, as early as embryonic day 7.5 (Ott and Bullock, 2001) and APP expression marginally increases during neurogenesis (Bergström et al., 2016; Arber et al., 2021). In adult mice, APP and APLP2 are expressed ubiquitously, while APLP1 is nervous system-specific (Lorent et al., 1995). APLP2 expression increases in disease associated microglia, suggesting a role in the disease process (Sala Frigerio et al., 2019). Excitatory neurons are known to express high levels of APP, most notably in the pyramidal cells of the cortex and hippocampus, however GABAergic interneurons also display expression (Wang et al., 2014; Hick et al., 2015).

### Cleavage

**FIGURE 2:**
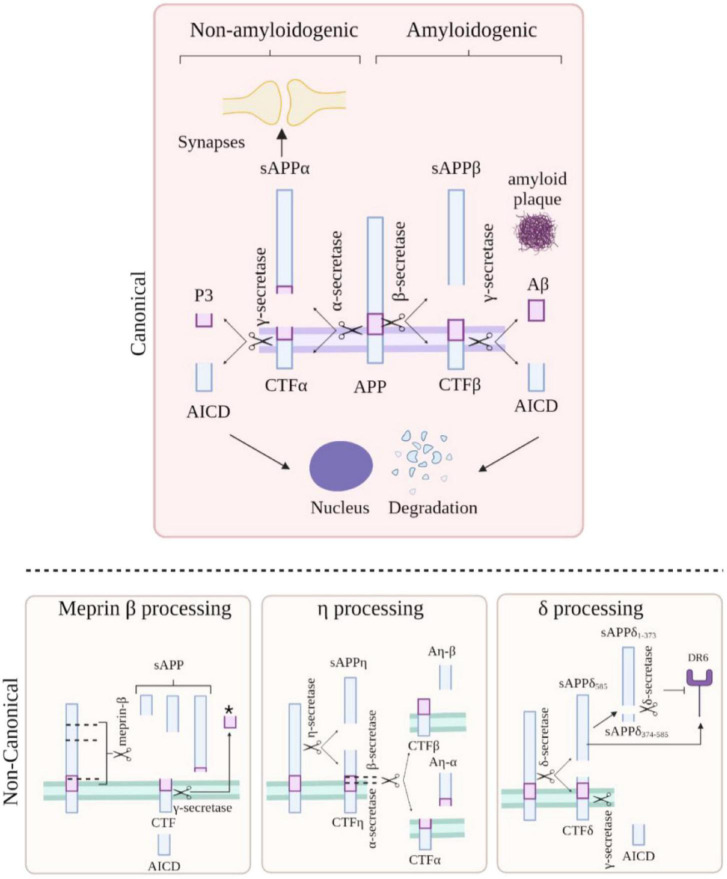
Amyloid precursor protein (APP) cleavage. APP can undergo canonical (top) and non-canonical (bottom) processing. In the amyloidogenic pathway (top, right side), APP is processed by β-secretase and γ-secretase resulting in the formation of Aβ peptides, APP intracellular domain (AICD) and sAPPβ. In the non-amyloidogenic pathway (top, left side), APP is cleaved by α-secretase and γ-secretase resulting p3 peptide, AICD, and sAPPα. Meprin-β cleavage (bottom left) generates three soluble APP fragments; the remaining CTF fragment can be further cleaved by γ-secretase giving rise to a smaller fragment indicated by * and AICD. APP cleavage by η-secretase (bottom, middle panel) generates an APP (sAPPη) and a CTFη fragment which can be further processed by either α or β-secretase and then by γ-secretase resulting in the formation of Aη-α/β and a CTFα or β fragment. APP cleavage by δ-secretase (bottom right) gives rise to a fragment which can activate the death cell receptor (DR6) promoting cell death or can be further cleaved to generate a fragment unable to bind the receptor; the remaining CTFδ fragment can be processed by γ-secretase form the intracellular domain AICD. Created with BioRender.com.

It is well-established that APP is cleaved by γ-secretase, α-secretases [including members of the A disintegrin and metalloproteinase (ADAM) family of proteins ADAM9, ADAM10, and ADAM17 (Allinson et al., 2003)] and by β-secretase 1 and β-secretase 2 (BACE1, BACE2) (Figure 2). However, several newly implicated secretases have also been recognised, such as cleavage of APP by η-secretase (Willem et al., 2015; Müller et al., 2017), Meprin-β and δ-secretase (Andrew et al., 2016). This results in many biologically active fragments of APP, some of which have been associated with AD pathogenesis.

Amyloidogenic processing of APP begins with β-secretase cleavage at the *N*-terminus of the Aβ sequence, which releases the soluble ectodomain fragment, sAPPβ. However, competitive cleavage along the non-amyloidogenic pathway by α-secretase is physiologically predominant. Stimulated by neuronal and synaptic activity, α-secretase cleavage occurs within the Aβ region of APP precluding Aβ peptide release and liberating the soluble ectodomain fragment, sAPPα (Gralle et al., 2006; Müller et al., 2017). At least 50% of the total forms of APP in the brain are constituted by sAPPα and sAPPβ (Morales-Corraliza et al., 2009). It is believed that α-secretase-based cleavage occurs at the plasma membrane, while β-secretase cleavage predominates in the endosomal compartments (Müller et al., 2017). sAPPα and sAPPβ differ only in the final 17 amino acids which correspond to a heparin-binding domain; absent in sAPPβ (Furukawa et al., 1996; Gralle et al., 2006; Peters-Libeu et al., 2015). Compelling evidence indicates that sAPPβ is less active that sAPPα and whether sAPPβ protein is stable or whether it undergoes further cleavage is still under debate (Nikolaev et al., 2009; Li et al., 2010).

Following cleavage by either α- or β-secretases, γ-secretase cleavage of the remaining C-terminal fragment releases either P3 or Aβ from the membrane (Tomita, 2014). Pathogenic mutations can modify this γ-secretase processing, destabilising the enzyme substrate interaction (Szaruga et al., 2017). This leads to release of longer, more aggregation prone species of Aβ, for example increasing the Aβ42 to Aβ40 ratio (Cacace et al., 2016; Arber et al., 2019; O’Connor et al., 2021).

### Post-translational Modification

APP can be post-translationally modified, influencing protein activity and increasing the diversity of APP species. Numerous ubiquitination sites have been described (Akimov et al., 2018) and there is evidence for Neddylation (Vogl et al., 2020); each with potential roles in protein stability.

Glycosylation is a further post-translational modification that can increase the diversity of APP function. Evidence suggests that cellular origin and disease status might affect the relative abundance of different glycosylated species of sAPPα and sAPPβ (Boix et al., 2020). This study suggests that different splice variants (APP695 vs. APP751/770) display different glycosylation moieties, potentially due to different cell origins, and that glycosylation of sAPPα differs between control and AD groups. Finally, palmitoylation is linked to APP localisation in lipid rafts and can affect downstream processing, favouring amyloidogenic processing (Bhattacharyya et al., 2021).

## Function

### Amyloid Precursor Protein and APLP Redundancy

**FIGURE 3:**
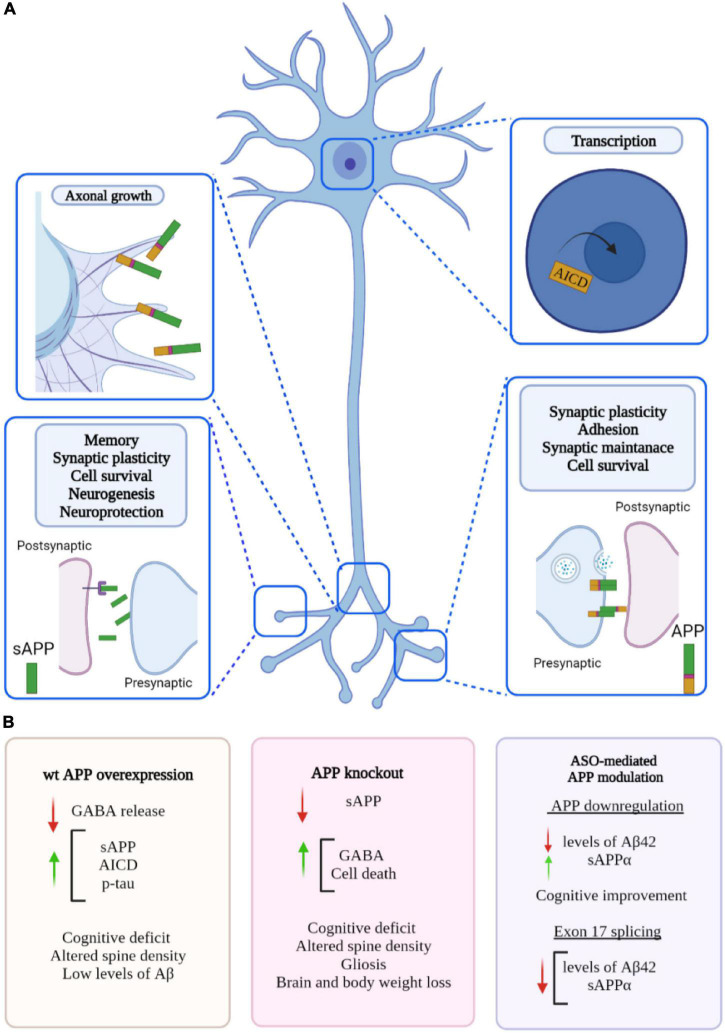
Proposed roles of amyloid precursor protein (APP) and phenotypes associated to changes in APP level. **(A)** APP plays a role in many biological processes including maintenance of synapse, transcriptional regulation, plasticity, and neuroprotection. APP is cleaved into biologically active fragments; APP intracellular domain (AICD) translocates to the nucleus where it regulates transcription; APP localises to the neuronal growth cone where it regulates axon growth; APP dimerization occurs at the synapse (in *trans* and in *cis*) between two molecules of APP regulating synaptic stability (similar dimerization occurs at the neuromuscular junction). **(B)** Phenotypes associated with overexpression of wild type APP, APP knockout and ASO-mediated APP modulation. Created with BioRender.com.

The APP protein family has many different physiological functions, including roles at the synapse, transcriptional regulation, plasticity and neuroprotection (Figure 3). APP can function as both a receptor and a ligand via its biologically active fragments, in particular sAPPα. Over 200 protein binding partners for APP have been identified (Müller et al., 2017) including extracellular proteins such as collagen and heparin, and soluble proteins such as spondin, the pancortins, and netrin. However, the functions of these interactions are not entirely clear (Müller et al., 2017). Precise understanding of these roles is critical to appreciate the impact of APP knockdown on neuronal homeostasis.

The APLPs have some overlapping functions with APP; knockout of single genes *in vivo* has only revealed subtle phenotypic differences, suggesting some level of functional compensation between APP, APLP1, and APLP2. However, dual knockout of APP/APLP2 and APLP1/APLP2 in mice has proved lethal, whilst APP/APLP1 knockout mice were apparently viable. This could indicate an important and distinct physiological role for APLP2 (Heber et al., 2000).

### Long Term Potentiation

Compelling evidence suggests that APP expression can regulate excitatory and/or inhibitory neurotransmission independent of Aβ plaque formation. Several studies have considered the ligand action of APP, in particular via its secreted sAPPα fragment. For example, sAPPα has been shown to bind to GABA type B receptors (Rice et al., 2019) and to regulate presynaptic neurotransmitter release, suggesting a role in inhibitory neuron physiology. Overexpression of wild-type human APP in mice causes early cognitive impairment and neuronal loss, without amyloid plaques (Mucke et al., 2000; Kreis et al., 2021). These mice exhibit neuronal overexcitation and increased long term potentiation. Whilst a decrease in GABA was detected, there was no change to GABA production or GABAergic receptor components, suggesting a link between sAPP fragments acting on presynaptic GABA type B receptors and inhibiting GABA release. This was further confirmed with use of a GABA type B receptor antagonist rescuing this overexcitation (Kreis et al., 2021). These findings suggest APP is vital for regulation of inhibitory neurotransmission.

In APP knockout mice (see Section Amyloid Precursor Protein Knockout Models), reintroduction of sAPPα either partially or entirely rescued deficits such as reduced brain weight, impaired long-term potentiation (LTP) and spatial learning (Ring et al., 2007). Altogether evidence suggests that sAPPα is crucial for mediating the physiological function of APP on synaptic plasticity. Secreted sAPPα fragments have been shown to bind to the cell surface transmembrane APP as an autocrine or paracrine ligand, triggering a G-protein signalling cascade which is vital for cell survival and neuroprotection (Milosch et al., 2014). *In vivo* expression of sAPPβ in the APP/APLP2 null mutant has no beneficial effects on postnatal lethality and fails to rescue neuromuscular synapse defects (Li et al., 2010), suggesting sAPPα is more functionally active than sAPPβ.

Amyloid precursor protein can also affect synaptic plasticity through Aβ production. Aβ itself has been shown to act as an activator of α7 nicotinic cholinergic receptors or a negative modulator, depending on the precise picomolar vs. nanomolar concentrations (Lasala et al., 2019; George et al., 2021).

Alternatively, cyclic guanosine monophosphate (cGMP) plays a significant role in LTP signal transduction, memory induction and maintenance. In mouse Neuro 2A cells, increasing cGMP induced a parallel increase in Aβ secretion. This was not due to an increase in APP expression, but rather there was an increase in the co-localisation of APP and BACE1, leading to additional amyloidogenic cleavage. Blocking Aβ function (either using antibodies or via APP knockout in mice) prevented cGMP-dependent enhancement of LTP and memory, suggesting this LTP improvement is dependent upon Aβ (Palmeri et al., 2017). This is especially interesting to consider, as elevated Aβ is of course associated with neurotoxicity in AD and has been shown to impair LTP (Hu et al., 2018).

A non-canonical pathway, via η-secretase cleavage of APP_695_, releases a truncated ectodomain fragment and a *C*-terminal fragment of higher molecular mass (CTF-η). Further cleavage of CTF-η by ADAM10 and BACE1 releases long and short Aη peptides, Aη-α and Aη-β. When BACE1 was inhibited in mice, CTF-η and Aη-α accumulated, reducing hippocampal LTP. Application of Aη-α *ex vivo* to hippocampal slices also lowered LTP (Willem et al., 2015), suggestive of negative regulation of LTP.

Amyloid precursor protein has also been shown to regulate GABA at the transcriptional level, further linking APP with LTP. In APP knockout neurons, decreased transcription of the inducible transcription factor neuronal PAS domain protein 4 (NPAS4) was observed. NPAS4 is regulated by neuronal depolarisation. Downregulation of NPAS4 occurred alongside an increase in GABA production. LTP measurements supported the increase in inhibitory neurotransmitter at the synapse in APP knockout mice (Opsomer et al., 2020).

### Transcription

Amyloid precursor protein may also play a role in the regulation of gene transcription. The APP intracellular domain (AICD), released during γ-secretase cleavage has been shown to interact with Fe65 which results in AICD stabilisation, nuclear translocation (Borg et al., 1996). Once in the nucleus, AICD can promote gene transcription (Cao, 2001). AICD may contribute to control of gene expression programs (Xinwei, 2001), such as cell signalling pathways, cytoskeletal changes (Müller et al., 2007) and negative feedback of APP and Aβ (Bukhari et al., 2017).

Amyloid precursor protein is also involved in the epigenetic regulation of immediate early genes involved in memory formation. APP was shown to regulate EGR-1 gene transcription both *in vitro* and *in vivo* (Hendrickx et al., 2013). Subsequent investigation into the expression of other immediate early genes involved in memory formation, such as c-Fos and BDNF, demonstrated further evidence of APP epigenetic mediation of gene expression (Hendrickx et al., 2014). Knockout of *APP* could therefore interrupt memory formation processes.

The impact of APP regulation of gene expression on physiological function has begun to be explored. After APP silencing in an *in vitro* nerve-muscle coculture, a reduction in both secreted glial cell line-derived neurotrophic factor (GDNF) and in the total number of neuromuscular junctions (NMJs) was observed, which was rescued when GDNF was subsequently expressed. Furthermore, expression of GDNF corrected the abnormal synaptic morphology of the NMJs in *APP* knockout mice (Stanga et al., 2016).

The fact that sAPPβ can regulate transcription of AICD target genes transthyretin (TTR) and Klotho in APP/APLP2 liver (Li et al., 2010) suggests that an APP signaling pathway could be involved in nuclear translocation and gene regulation.

### Receptor and Ligand Action

Many potential ligands for APP have been identified, including F-spondin, Reelin, B1 integrin, Lingo-1, and Pancortin-1 (Rice et al., 2013). However, their roles are somewhat unclear. In a study to identify whether any of these ligands could stimulate and regulate α- and β-secretase cleavage of APP, it was found that Reelin, Lingo-1, and Pancortin-1 appear to inhibit the shedding of the ectodomain fragments (Rice et al., 2013). Based on the role of these ligands, it appears that APP has an important role in axonal pathfinding and cell attachment (for example at the synapse and at the NMJ).

### Axonal Outgrowth

Amyloid precursor protein has been shown to localise to neuronal growth cones as well as synaptic boutons (Sabo et al., 2001; Young-Pearse et al., 2008). Knockout of APP inhibits axonal outgrowth *in vitro* (Sosa et al., 2017). This suggests APP expression is required for axonal sprouting and organisation and may impact axodendritic connectivity and neuronal activity (Deyts et al., 2016).

Presenilin-1 (PSEN1) is an essential component of γ-secretase, and pathogenic mutations in PSEN1 increase the production of more neurotoxic Aβ in familial AD (Chávez-Gutiérrez et al., 2012; Arber et al., 2020). Neurons with a *PSEN1* mutation have been shown to exhibit aberrant axodendritic outgrowth, due to increased levels of the intracellular APP C-terminal fragments (Deyts et al., 2016).

Recently reported patients harbouring homozygous non-sense mutations in APP display microcephaly and a reduced corpus callosum, supporting a role of APP in axonal outgrowth (Klein et al., 2016).

### Cell Adhesion and Roles at the Synapse

Amyloid precursor protein can form both *cis* and *trans* dimers, which can be homophilic (APP+APP) or heterophilic (APP+APLP1/2). Trans-dimerisation allows extracellular cell-cell adhesion, and the formation of such terminal fragments, suggesting the ectodomain is important for this process. Moreover, removal of the APP intracellular domain strongly increases APP dimerization. The processing of APP has been shown to be influenced by APP dimerization, whereby increased APP dimerization is linked with increased non-amyloidogenic processing (Decock et al., 2015).

Amyloid precursor protein also plays a role in the development and function of the neuromuscular junction (NMJ). Transgenic mice with reduced or eradicated APP expression have demonstrated synaptic deficits, including compromised neurotransmitter release and impaired organisation of post-synaptic receptors at the NMJ (Caldwell et al., 2013).

Furthermore, deletion of both APP and APLP2 causes impaired neurotransmitter release. This suggests that APP and APLP2 facilitate transmitter release through interaction at presynaptic sites (Fanutza et al., 2015). However, the functional compensation between APP and the APLPs could mean knockout of one single gene might not elicit the same impairments.

### Additional Putative Functions of Amyloid Precursor Protein

A number of other potential roles of APP have been described (Müller et al., 2017). These include roles of the secreted forms of APP in nueurogenesis (Caillé et al., 2004), and neuroprotection (Corrigan et al., 2014); for example, while sAPPα can neutralize Aβ toxic effects on neurite outgrowth *in vitro*, sAPPβ at the same concentration, has no effect (Li et al., 1997). Higher concentration of sAPPβ seems to promote neuronal survival (Yamamoto et al., 1994) and neurite extension in B103 cells (Jin et al., 1994), altogether suggesting sAPPβ is less active compared to sAPPα.

Amyloid precursor protein is also present in the endo-lysosome system and patient models of familial AD show swollen endosomes (Israel et al., 2012) and altered autophagy (Hung and Livesey, 2018). Furthermore, APP is found at the mitochondrial-ER contact sites suggesting a role in bioenergetics (Schon and Area-Gomez, 2013).

Finally, in addition to studies into its toxicity, Aβ itself has been proposed to have physiological functions, for example in fibrilization and virus entrapment akin to opsonisation (Eimer et al., 2018). This hypothesis is supported by evidence that certain herpesviruses are overrepresented in AD post-mortem tissue (Readhead et al., 2018).

### Concluding Remarks

Characteristic spatiotemporal expression, splicing, cleavage, and post-translational modification all result in a diversity of APP-derived species. Presumably as a result of this diversity, APP has been implicated in a wide range of cellular functions.

The rationale for reducing Aβ in AD appears convincing. However, the impact of lowering *APP* expression, and the concurrent reduction of APP species besides Aβ, requires careful consideration.

To understand the consequence of APP reduction strategies, a detailed knowledge of the function of APP is paramount. To this end, APP knockout models have been extensively studied to infer the major consequences of loss of APP function.

## Amyloid Precursor Protein Knockout Models

The generation of *in vitro* and *in vivo* APP knockout (KO) models has provided insights into the physiological role of APP. For a detailed list of available KO models see reviews such as Senechal et al. (2006) and Müller et al. (2017). Two APP KO mice have been extensively characterised, a homozygous APP null mutant (Zheng et al., 1995) and an APP deficient mouse (APPD) in which only 5% of normal APP is expressed (the majority of APP transcripts represent a shorter form due to a deletion of APP exon 2) (Muller et al., 1994). Other *in vivo* KO models include *Drosophila* lacking APPL (Luo et al., 1992; Torroja et al., 1999; Gunawardena and Goldstein, 2001) and *Caenorhabditis elegans* lacking *apl-1* (Hornsten et al., 2007), both orthologues of human APP.

Amyloid precursor protein KO mice and flies are viable and fertile (Luo et al., 1992; Muller et al., 1994; Zheng et al., 1995); however, lack of APPL in *Drosophila* leads to shorter lifespan (Wentzell et al., 2012) while loss of *apl-1* in *C. elegans* results in larval lethality (Hornsten et al., 2007). Nevertheless, in mice, APP-family members have redundant functions and may play a compensatory role (see Section Amyloid Precursor Protein and APLP Redundancy). In line with this, mice double or triple KO for APP/APLP2 or APP/APLP2/APLP1 display postnatal lethality (von Koch et al., 1997; Heber et al., 2000).

Although APP is not essential for survival, APP KO mice are associated with hypoactivity and reduced grip strength underlying muscular and/or neuromuscular defects (Muller et al., 1994; Zheng et al., 1995). Moreover, these mutant animals are characterised by reduced body and brain weight (Zheng et al., 1995; Magara et al., 1999). Interestingly, depending on the genetic background, lack of APP can result in neuroanatomical abnormalities including agenesis of the corpus callosum and hippocampal commissure defects (Muller et al., 1994; Zheng et al., 1995; Magara et al., 1999).

The absence of APP in mice, mainly affects the hippocampus and cortex where it is possible to observe an altered distribution of dendritic marker MAP2 and presynaptic marker synaptophysin (Zheng et al., 1995; Dawson et al., 1999; Seabrook et al., 1999), as well as reduction of dendritic length and projection depth in the CA1 hippocampal neurons (Dawson et al., 1999; Seabrook et al., 1999). Both neuronal marker loss and aberrant morphology are linked to disruption of synaptic functioning and synaptic plasticity. Accordingly, loss of APP impairs long-term potentiation (LTP) in the hippocampus of mutant mice (Dawson et al., 1999; Seabrook et al., 1999; Fitzjohn et al., 2000).

Consistent with altered synaptic plasticity, the absence of APP has been associated with reduced synaptic vesicle density and smaller active zone (Phinney et al., 1999; Yang et al., 2005). The role of APP in synaptic maintenance is further supported by the reduction of neuromuscular junctions in *Drosophila* null mutants lacking APPL (Torroja et al., 1999). Moreover, APP KO mice perform worse in conditioned avoidance (Muller et al., 1994; Dawson et al., 1999; Senechal et al., 2006) and Morris water maze tasks (Muller et al., 1994; Sugaya et al., 1996; Tremml et al., 1998; Phinney et al., 1999) therefore displaying deficits in learning and memory formation, underlying a key role for APP in cognitive functions.

Amyloid precursor protein knockout mice display astrogliosis in the hippocampus and throughout the cortex (Zheng et al., 1995; Dawson et al., 1999; Seabrook et al., 1999). However, loss of APP in the substantia nigra has been shown to have a neuroprotective effect following lesions of this area, possibly by avoiding the formation of APP *C*-terminal fragments and by reducing microglial activation (de Giorgio et al., 2002).

As mentioned above, a rare case of an individual carrying a homozygous non-sense mutation has been reported, representing a complete APP knockout (Klein et al., 2016). These human “examples” can be very informative. Interestingly, loss of APP in this individual was associated with consistent phenotypes with those observed in the APP KO murine models, including decreased body and brain weight, an abnormal corpus callosum, and decreased locomotor activity.

*In vivo* KO studies have highlighted the importance of APP during development and implicated physiological functions, such as in regulating synaptic plasticity and cognition. Of note, in the absence of an APP conditional model, we are unable to determine the biological consequences of APP KO in an adult setting. Altogether, APP KO models might not be the most appropriate system to explore APP reduction as a potential disease-modifying strategy.

## Amyloid Precursor Protein Downregulation as a Therapeutic Target

### Therapeutic Progress to Date

To explore the relevance of APP reduction as a therapy for AD, acute APP knockdown/knockout has been performed *in vivo* and *in vitro* using a variety of techniques including antisense oligonucleotides, small interfering RNAs (siRNAs) and CRISPR/Cas9 gene editing in both wild type and disease models. In this review, we will focus on the effect of APP downregulation in AD-associated models.

It is clear that increased APP dosage is a definitive risk factor for AD. Increased APP is associated with increased Aβ production and amyloid plaque formation. Over the past decades Aβ has been a major therapeutic target. Previous therapeutic strategies have modulated APP cleavage using small molecule inhibitors or modulators of γ- and β-secretase.

Several compounds have been developed and only a few have entered later stage clinical trials. Semagacestat and Avagacestat, two γ-secretase inhibitors have shown to successfully reduce Aβ production, however important side effects raised concern about the safety of these small molecules (Coric et al., 2012; Doody et al., 2013). Off target effects are inevitable, due to the large range of substrates recognised by γ-secretase (Haapasalo and Kovacs, 2011). Additionally, the aberrant accumulation of APP *C*-terminal fragments is thought to be cytotoxic (Mitani et al., 2012). Therefore, development of γ-secretase modulators that selectively affect APP processing rather than non-specific γ-secretase inhibitors represent a more favorable approach. E2212 is an example of a γ-secretase modulator that entered clinical trials (Yu et al., 2014).

Similar to γ-secretase, development of β-secretase inhibitors has been quite challenging, and a number of trials have been terminated due to worsening of clinical measurements of disease (Yan and Vassar, 2014). Although small molecule modulators to γ-secretase and β-secretase inhibitors have the potential to become therapeutic treatments for AD, clinical trials of these small molecules have revealed limitations.

Aβ immunotherapy has also been explored as a potential approach to reduce Aβ burden, with a number of antibodies at various stages of the clinical trial pipeline. Examples of Aβ immunotherapies include bapineuzumab, solanezumab, gantenerumab, and aducanumab (Penninkilampi et al., 2017). Aducanumab has had accelerated approval by the FDA and it is now available in the United States; there are however concerns about its clinical efficacy and it has not been approved by the European Medicine Agency (EMA).

New therapeutic avenues are emerging which aim to lower Aβ formation by acting directly at the level of the DNA or RNA of APP. Antisense oligonucleotides (ASOs) represent one of these newer approaches. An *in vitro* study, showed that reducing the APP substrate concentration available for γ-secretase was sufficient to reduce the ratio of Aβ42 relative to Aβ40, suggestive of a more complete enzymatic cleavage and reduced levels of toxic Aβ species (Ye et al., 2007). Moreover, loss of APP in mice and humans have only subtle phenotypes, therefore APP downregulation could have beneficial effects without affecting viability. Several ASOs aiming to modulate APP expression have been generated to treat AD (see Table 1).

**TABLE 1:** Summary of antisense oligonucleotide (ASO) knockdown studies to date.

ASO	Target	Mechanism of action	*In vitro* testing	*In vivo* testing	References
OL-1	APP mRNA	RNA degradation	-	SAMP8 and Tg2576 mice	Kumar et al., 2000; Farr et al., 2014
SSO	APP exon 17 or 15 in mice	Exon skipping	DS fibroblast	C57BL/6J Mice	Chang et al., 2018
AON	APP exon 17 (or 15 for mice)	Exon skipping	HCHWA-D fibroblast	C57BL/6J Mice	Daoutsali et al., 2021
ODN	APP site near beta secretase cleavage	RNA degradation	APP/Swe fibroblasts	Tg2576 mice	Chauhan and Siegel, 2007

### Amyloid Precursor Protein Downregulation Using Antisense Oligonucleotides

Over the past few years, antisense oligonucleotides (ASOs) have proved to be powerful therapeutic tools. These RNA-based therapeutics aim to alter the expression of a target gene by binding to a specific RNA molecule according to Watson and Crick base pairing. ASOs are synthetic single-stranded DNA molecules that recognizes both coding and non-coding RNA molecules and can promote RNA degradation, inhibit translation, or modulate RNA splicing. A detailed description of the ASOs mechanism of action can be found in recent reviews (Crooke et al., 2021a,b). Several ASOs aiming to modulate APP expression have been generated to treat AD (see Table 1).

OL-1, an antisense oligonucleotide against APP, successfully reduces APP levels in AD mice models Tg2576 and SAMP8 (Kumar et al., 2000; Farr et al., 2014). Tg2576 mice overexpress human APP carrying the Swedish mutation (KM670/671NL) and are characterised by increased Aβ levels and significant deposition of Aβ into plaques (Hsiao et al., 1996) while SAMP8 mice are characterised by age-associated increase of murine Aβ in the hippocampus (Yagi et al., 1989). Nevertheless, both models are characterised by learning and memory impairment, oxidative stress, and neuroinflammation (Kumar et al., 2000; Farr et al., 2014). Although OL-1 fails to lower soluble Aβ levels, it effectively improves cognition and reduces neuroinflammation in both models (Kumar et al., 2000; Farr et al., 2014).

Delivery of an ASO inducing translational blocking of APP transcript near the β-cleavage site (ODN), in Tg2576, successfully reduced Aβ levels, while promoting α-secretase cleavage over β-secretase cleavage (Chauhan and Siegel, 2007). Interestingly, administration of ODN results in an increased production of sAPPα which could have important consequences considering the several biological functions associated to this APP fragment.

Another ASO has been designed to promote APP exon 17 skipping (SSO), resulting in the generation of an APP isoform lacking the γ-secretase cleavage site and therefore unable to produce Aβ peptides (Chang et al., 2018). Transfection of the SSO in Down Syndrome (DS) patient-derived fibroblasts, leads to a dose-dependent decrease of full-length *APP* and results in reduced and normalised levels of the Aβ42 peptide (Chang et al., 2018). The same phenotype was observed *in vivo* following the post-natal delivery of the ASOs in DS and wild type mice models in the hippocampus and cortex. Importantly, ASO treatment neither affected the weight of the mice nor did it induce gliosis, observed in APP KO animals, however *in vitro* evidence suggests that the newly synthesised protein escapes α and β processing and therefore does not result in the formation of a CTF nor sAPP fragments (Chang et al., 2018).

Antisense oligonucleotide-mediated skipping of *APP* exon17 has been developed for Hereditary cerebral haemorrhage with amyloidosis-Dutch type (HCHWA-D), a disease caused by a mutation at codon 693 of APP; near to the α-secretase cleavage site. Similar to SSO, this ASO (AON) significantly reduces the APP level in favour of APP isoform lacking exon 17. This was shown both at the RNA and protein level in iPSCs derived cell lines, in fibroblasts from HCHWA-D affected individuals as well as *in vivo* in wild type mice (Daoutsali et al., 2021). Interestingly, in these models both Aβ40 and Aβ42 species were significantly reduced following administration of the ASO *in vitro* and *in vivo* (Daoutsali et al., 2021); however, contrarily to what has been observed by Chang et al. (2018), different brain areas display different degrees of exon skipping (Daoutsali et al., 2021). Moreover, although exon 17 skipping does not affect the α-secretase cleavage site, less sAPPα is observed following AON administration (Daoutsali et al., 2021). On top of that, possible side effects of generating a novel, non-physiological form of APP needs to be addressed.

Overall, downregulation of APP mRNA or modulation of the splicing of the exon containing the γ- secretase cleavage site appears to result in potentially beneficial outcomes with respect to Aβ biology, without repercussions on cell viability. Nevertheless, modulation of APP alters the generation of all its cleavage products, and although Aβ is considered the main culprit in AD, the impact of reducing other bioactive species is an important consideration.

### Modulating Amyloid Precursor Protein Expression via CRISPR/Cas9 Genome Editing

Another potential disease-modifying therapy is CRISPR/Cas9 gene editing. CRISPR technology has acquired popularity over the past few years and has become the most widely used strategy for genome editing. The CRISPR/Cas9 system is based on an interaction between an RNA guide and target DNA sequence, which as for the ASOs, is based on a Watson and Crick base pairing. A detailed description of the CRISPR/Cas9 mechanism of action can be found in recently published review (Wang et al., 2016). The CRISPR system has been used successfully and specifically to correct APP mutations in human APPswe fibroblasts, leading to a marked reduction of Aβ40 and Aβ42 species (György et al., 2018). The same is true for correcting APP mutations in iPSC-derived neurons (Kwart et al., 2019). *In vivo*, although delivery of CRISPR/Cas9 successfully targets the APPswe allele in Tg2567 mice, only about 2% of the mutated allele is disrupted.

The therapeutic relevance of APP reduction strategies has been reinforced by CRISPR/Cas9 knockout of APP in iPSC models. Mutations in *PSEN1* cause autophagic and lysosomal dysfunction (Peric and Annaert, 2015; Hung and Livesey, 2018) and the CRISPR/Cas9-mediated knockout of APP is sufficient to reverse these phenotypes (Hung and Livesey, 2018).

### siRNA Mediated Amyloid Precursor Protein Knockdown

siRNA mediated APP knockdown has also been tested *in vivo*. Acute APP knockdown successfully downregulated APP mRNA in C57BL/6JIco mice brain, especially in the CA2-CA3 regions of the hippocampus (Senechal et al., 2007). Contrarily to APP KO models, no altered forelimb grip strength or locomotor activity was observed; however, siRNA infused mice displayed deficits of spatial working memory (Senechal et al., 2007). In the future, it would be interesting to look at siRNA mediated APP KO in AD models that overexpress APP or carry AD-associated mutations in *APP*.

## Discussion

### Biological Consequences of Amyloid Precursor Protein Reduction

Amyloid precursor protein plays important roles in synaptic plasticity, cell adhesion and other neuronal and non-neuronal functions, many of which remain unknown. Complex APP processing leads to numerous active fragments whose functions are diverse and largely unclear. This brings up an important issue: what are the biological consequences of downregulation of each these APP species?

Further investigation is necessary to respond to this question. So far studies have highlighted that ASO-mediated downregulation ultimately leads to a reduction of Aβ *in vitro* and *in vivo*. Importantly, *in vivo* reduction of Aβ is associated with memory improvement (Kumar et al., 2000) and less cytotoxicity (Farr et al., 2014). Chauhan and Siegel (2007) showed that ASO-mediated APP downregulation resulted in a 40% increase in sAPPα. Evidence suggests that APP overexpression and downregulation have similar impacts on synaptic plasticity, meaning sAPPα dosage requires careful consideration. However, sAPPα has primarily been associated with neuroprotective functions and reintroduction of sAPPα either partially or entirely rescued deficits in APP knockout mice; such as reduced brain weight, impaired LTP and spatial learning (Ring et al., 2007).

APP exon skipping (exon 15 in mice and 17 in human cells) generates a new non-physiological isoform lacking the γ-secretase domain. This results in lower Aβ levels but also in a reduction of sAPPα and sAPPβ (Chang et al., 2018; Daoutsali et al., 2021). This exon skipping event leads to the disruption of the transmembrane domain and the generation of a new soluble, secreted form of APP (Chang et al., 2018). This could have important biological consequences not only due to the reduction of membrane-associated APP but also due to putative gain of toxic functions associated with this new species.

Lastly, therapeutic knockdown of APP will affect the post-developmental roles of APP. Information from knockout models of APP inform on its essential roles. However, in the absence of a conditional APP knockout model, the exact consequence of APP reduction in adult cells is difficult to determine.

### Therapeutic Opportunities

Despite many potential therapeutic approaches having been tested for AD, the search for effective disease-modifying therapies remains elusive. Increased expression of APP leads to Alzheimer’s disease, where Aβ directly contributes to pathologies. For example, three copies of *APP* in Down’s Syndrome (Wisniewski et al., 1985) as well as local duplications (Rovelet-Lecrux et al., 2006; Sleegers et al., 2006) and triplications (Grangeon et al., 2021) are causative of dementia.

For these reasons, *APP* reduction strategies are attractive for AD, potentially limiting pathological protein accumulation and thereby disease and clinical progression. It should be noted that late onset AD is likely to be mechanistically complex and it is less clear whether APP reduction can impact other disease processes such as tau aggregation, neuroinflammation and subsequent neurodegeneration.

Amyloid precursor protein knockdown appears safe, as people living with one copy of APP are asymptomatic (Klein et al., 2016). Complete APP knockout in both mice and humans, although not lethal, is associated with relatively subtle phenotypes. These include reduced brain weight, gliosis and deficits in synaptic biology (Muller et al., 1994; Zheng et al., 1995), effects shared in the one human case of complete APP loss of function (Klein et al., 2016).

Nevertheless, ASO and CRISPR technologies do not lead to total knockout of APP, representing partial reduction therapies. On top of that, APLP2 shows a compensatory role in the absence of APP; demonstrated as APP/APLP2 double KO models are unviable, contrary to APP knockout alone, further strengthening the potential of an APP reduction-based therapy.

These new approaches seem to be specific and safe overcoming some limitations associated with β and γ-secretase inhibitors. Importantly, from 2016 to 2020, the FDA has approved three ASO-based therapies for patients with Duchenne muscular dystrophy (DMD). Although it is too early to be able to draw conclusions on ASO-based AD therapies, the development of safe and clinically effective ASO-mediated therapies for other diseases raises hopes for AD.

In conclusion, more complete knowledge of the function of APP and the consequence of reducing its expression are required. Challenges remain, such as delivery of genetic therapies. However, a 50% increase in *APP* expression (via *APP* duplication) brings forward the predicted age of onset by at least 30 years. It is tantalising to predict the effect that small reductions in APP expression might have on familial AD mutation carriers, but also more widely for those at risk of, or in the early stages of, AD.

## Author Contributions

RG, EA, and CA: writing original draft. All authors writing review and editing, contributed to the article, and approved the submitted version.

## Conflict of Interest

The authors declare that the research was conducted in the absence of any commercial or financial relationships that could be construed as a potential conflict of interest.

## Publisher’s Note

All claims expressed in this article are solely those of the authors and do not necessarily represent those of their affiliated organizations, or those of the publisher, the editors and the reviewers. Any product that may be evaluated in this article, or claim that may be made by its manufacturer, is not guaranteed or endorsed by the publisher.
